# Beyond the Common: A Case Report on Right Paraduodenal Hernia

**DOI:** 10.7759/cureus.52723

**Published:** 2024-01-22

**Authors:** Thikra M Alblowi

**Affiliations:** 1 General Surgery, Royal Commission Medical Center, Yanbu, SAU

**Keywords:** mesenteric defect, internal hernia, laparoscopic hernia repair, intestinal obstruction, right paraduodenal hernia

## Abstract

Paraduodenal hernias, particularly those on the right side, are rare but clinically significant occurrences characterized by the abnormal protrusion of abdominal contents through mesenteric defects adjacent to the duodenum. These hernias result from embryologic malformations and can lead to complications such as intermittent abdominal pain, nausea, and, in severe cases, bowel obstruction. This case describes a 48-year-old male who presented with a 24-hour history of colicky abdominal pain in the right upper quadrant, associated with nausea. Further investigation, including a computed tomography scan, revealed a right paraduodenal hernia with herniation of small bowel loops through a mesenteric defect. Timely surgical intervention via laparoscopic exploration confirmed the diagnosis and facilitated the reduction of herniated bowel, followed by meticulous repair of the mesenteric defect using nonabsorbable sutures. The patient's recovery was uneventful, with a return to normal bowel function, and postoperative follow-up showed the resolution of symptoms. This case underscores the clinical complexity and management challenges associated with right paraduodenal hernias. Surgical intervention, guided by laparoscopic exploration, emerged as an effective and minimally invasive approach. The successful reduction of herniated small bowel loops and meticulous closure of the mesenteric defect contributed to a favorable postoperative course, highlighting the importance of timely intervention to prevent complications.

## Introduction

Paraduodenal hernias are infrequent but clinically significant entities characterized by the abnormal protrusion of abdominal contents through mesenteric defects adjacent to the duodenum [1]. Among these, right paraduodenal hernias represent a distinct subset, accounting for a minority of cases [1,2]. These hernias result from an embryologic malformation where the midgut fails to rotate adequately, creating potential spaces for herniation within the mesentery. While often asymptomatic, they can lead to intermittent abdominal pain, nausea, and, in severe cases, bowel obstruction [1,2].

The diagnostic challenge lies in the rarity of these hernias and the subtle nature of their clinical presentation [2]. Accurate identification typically requires a combination of clinical acumen and imaging studies, with computed tomography playing a pivotal role in confirming the diagnosis [2]. This case presentation explores the details of a 48-year-old male diagnosed with a right paraduodenal hernia.

## Case presentation

A 48-year-old male presented to the emergency department with a 24-hour history of intermittent abdominal pain in the right upper quadrant. The pain was described as colicky and associated with nausea but no vomiting. The patient denied any recent changes in bowel habits or urinary symptoms. His medical history was notable for hypertension, for which he was taking an angiotensin-converting enzyme inhibitor.

Upon initial examination, the patient appeared uncomfortable but not acutely distressed. Vital signs were within normal limits, and physical examination revealed tenderness in the right upper quadrant of the abdomen with no rebound or guarding. Bowel sounds were present and normal. Digital rectal examination was unremarkable.

Given the persistent nature of the abdominal pain, further investigation was initiated. Laboratory investigations revealed a normal complete blood count, liver function tests, and pancreatic enzyme levels. Serum electrolytes and renal function were within normal limits (Table 1).

**Table 1:** Laboratory investigation results upon admission.

Laboratory test	Result	Reference range
Hemoglobin	13.5 g/dL	13.5-17.5 g/dL
White blood cell count	8,000 cells/mm³	4,000-11,000 cells/mm³
Platelet count	250,000 cells/mm³	150,000-450,000 cells/mm³
Alanine aminotransferase	25 U/L	7-56 U/L
Aspartate aminotransferase	30 U/L	15-41 U/L
Alkaline phosphatase	75 U/L	44-147 U/L
Total bilirubin	0.8 mg/dL	0.2-1.2 mg/dL
Direct bilirubin	0.3 mg/dL	0.0-0.3 mg/dL
Amylase	60 U/L	30-110 U/L
Lipase	40 U/L	23-300 U/L
Sodium	140 mEq/L	135-145 mEq/L
Potassium	4.0 mEq/L	3.5-5.0 mEq/L
Chloride	100 mEq/L	98-107 mEq/L
Bicarbonate	26 mEq/L	22-29 mEq/L
Blood urea nitrogen	20 mg/dL	7-20 mg/dL
Creatinine	1.0 mg/dL	0.6-1.3 mg/dL

**Figure 1:**
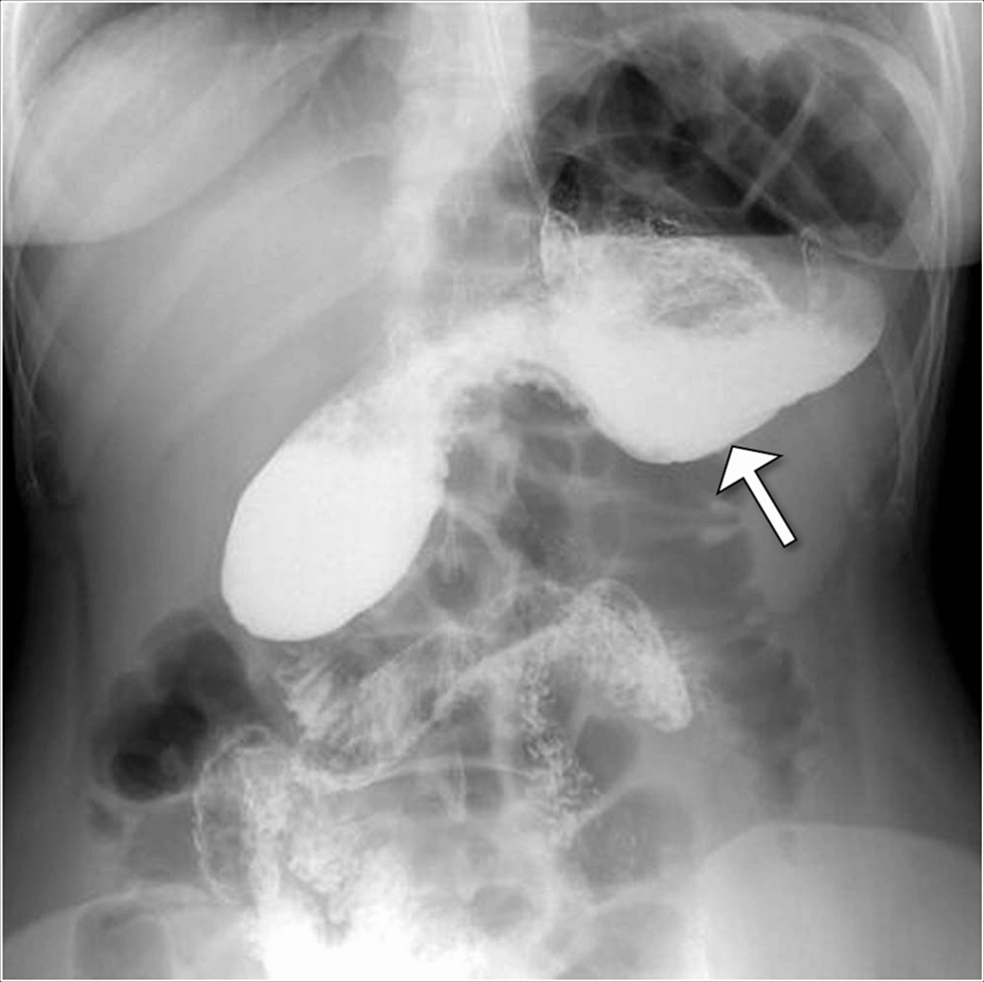
Barium upper gastrointestinal study illustrating delayed gastric emptying (arrow).

**Figure 2:**
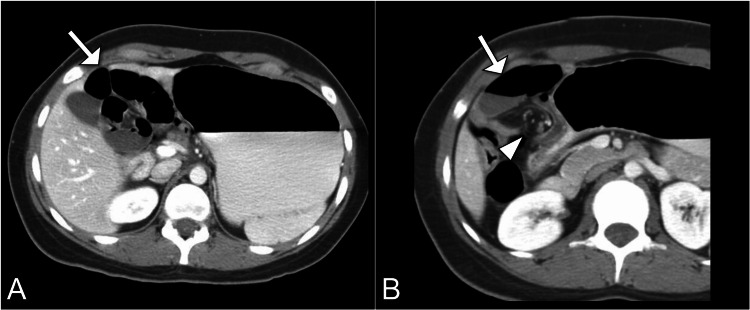
Axial CT images reveal a cluster of dilated jejunal loops (arrow) in the right upper quadrant (A) and a twist in the blood vessels (arrowhead) (B), indicative of a right paraduodenal hernia. CT, computed tomography

A barium upper gastrointestinal study showed delayed gastric emptying (Figure 1). Subsequently, a computed tomography scan of the abdomen and pelvis with contrast was performed, revealing a cluster of dilated jejunal loops in the right upper quadrant through a defect in the mesentery located posterior to the ascending colon (Figure 2).

The patient was admitted to the surgical service, and a consultation with the general surgeon was obtained. The surgeon corroborated the diagnosis based on clinical and radiological findings. After adequate preoperative preparation, the patient underwent an elective laparoscopic exploration. Intraoperatively, a right paraduodenal hernia was confirmed, with the incarceration of small bowel loops. The herniated bowel was carefully reduced, and the defect in the mesentery was repaired using interrupted nonabsorbable sutures.

Postoperatively, the patient's recovery was uneventful. He tolerated a regular diet, and bowel movements were resumed within the first 48 hours after surgery. Pain control was achieved with oral analgesics, and he was discharged on the third postoperative day with instructions for outpatient follow-up.

The patient was seen in the outpatient clinic two weeks after discharge, and he reported a complete resolution of the abdominal pain. The surgical incisions were well-healed, and there were no signs of infection or hernia recurrence on physical examination.

## Discussion

The presented case of a right paraduodenal hernia underscores the clinical complexity and management challenges associated with this rare condition. Paraduodenal hernias, although infrequently encountered, demand attention due to their potential for serious complications, including bowel obstruction and ischemia [1,2]. Our discussion delves into several key aspects, including the diagnostic approach, surgical considerations, and postoperative management, emphasizing the relevance of this case in contributing to the existing medical literature.

Accurate diagnosis of paraduodenal hernias is often elusive, given their nonspecific clinical presentation [3]. In our case, the patient's intermittent abdominal pain and associated nausea prompted further investigation, culminating in a timely computed tomography scan that revealed the herniated small bowel loops. The diagnostic challenge lies in the rarity of these hernias and the potential for delayed recognition, which can result in complications such as bowel obstruction or strangulation [2-4]. This underscores the importance of considering paraduodenal hernias in the differential diagnosis of patients presenting with unexplained abdominal pain, particularly when located in the right upper quadrant [2,3].

Surgical intervention remains the cornerstone of management for symptomatic paraduodenal hernias [2,3]. The decision between open and laparoscopic approaches depends on the surgeon's expertise and the clinical presentation. In our case, a laparoscopic exploration facilitated a thorough examination, confirmed the diagnosis, and allowed for a minimally invasive repair of the mesenteric defect [3-5]. This approach aligns with the evolving trend towards less invasive procedures, offering reduced postoperative pain, shorter hospital stays, and faster recovery [4,5].

A critical consideration in the surgical management of paraduodenal hernias is the prevention of recurrence [2,4]. In our case, the meticulous repair of the mesenteric defect using nonabsorbable sutures aimed to minimize the risk of hernia recurrence. Postoperatively, the patient's uneventful recovery and prompt return to normal bowel function underscore the effectiveness of the surgical intervention.

## Conclusions

While paraduodenal hernias are rare, their clinical significance and potential for complications necessitate increased awareness among clinicians. This case contributes to the limited body of literature on right paraduodenal hernias, providing insights into the diagnostic approach, surgical considerations, and postoperative management. Further research and case reports are warranted to enhance our understanding of this condition, refine diagnostic algorithms, and optimize treatment strategies. This case underscores the value of a multidisciplinary approach involving surgeons, radiologists, and gastroenterologists in managing these complex abdominal pathologies.
